# Gene expression and immunohistochemical analyses of mKast suggest its late pupal and adult-specific functions in the honeybee brain

**DOI:** 10.1371/journal.pone.0176809

**Published:** 2017-05-04

**Authors:** Atsuhiro Yamane, Hiroki Kohno, Tsubomi Ikeda, Kumi Kaneko, Atsushi Ugajin, Toshiyuki Fujita, Takekazu Kunieda, Takeo Kubo

**Affiliations:** Department of Biological Sciences, Graduate School of Science, The University of Tokyo, Bunkyo-ku, Tokyo, Japan; University of Cologne, GERMANY

## Abstract

In insect brains, the mushroom bodies (MBs, a higher center) comprise intrinsic neurons, termed Kenyon cells (KCs). We previously showed that the honeybee (*Apis mellifera* L.) MBs comprise four types of KCs, in addition to the previously known three types of KCs: class I large-type KCs (lKCs), class I small-type KCs (sKCs) and class II KCs, novel class I ‘middle-type’ KCs (mKCs), which are characterized by the preferential expression of a gene, termed *mKast*. Although *mKast* was originally discovered during the search for genes whose expression is enriched in the optic lobes (OLs) in the worker brain, subsequent analysis revealed that the gene is expressed in an mKC-preferential manner in the MBs. To gain more insights into the function of mKast in the honeybee brain, we here performed expression analysis of *mKast* and immunohistochemistry of the mKast protein. Prominent *mKast* expression was first detected in the brain after the P7 pupal stage. In addition, *mKast* was expressed almost selectively in the brain, suggesting its late pupal and adult specific functions in the brain. Immunohistochemistry revealed that mKast-like immunoreactivity is detected in several regions in the worker brain: inside and around the MB calyces, at the outer edges of the OL lobula, at the outer surface of and posterior to the antennal lobes (ALs), along the dorsal midline of the anterior brain and at the outer surface of the subesophageal ganglions (SOG). mKast-like immunoreactivities in the MBs, OLs, ALs and SOG were due to the corresponding neurons, while mKast-like immunoreactivities beneath/between the MB calyces were assumed to most likely correspond to the lateral/medial neurosecretory cells.

## Introduction

The European honeybee (*Apis mellifera* L.) is a eusocial insect and their colony members exhibit various exquisite social behaviors, including the well-known dance communication [1–3]. The detailed neural bases of their social behaviors, however, are still not well understood. Among other compartments, insect brains comprise the mushroom bodies (MBs; higher order processing centers), optic lobes (OLs; visual centers), antennal lobes (ALs; olfactory centers), and subesophageal ganglion (SOG), a center for sensory and motor processing related to mouthparts functions [4–10]. The honeybee MBs are paired brain structures and each MB has two cup-like structures, termed calyces. Previous studies have suggested that the honeybee MBs comprise three types of KCs, intrinsic MB interneurons: class I large-type KCs (lKCs, also termed ‘class I non-compact KCs’) and class I small-type KCs (sKCs, also termed ‘class I compact KCs’), whose somata are localized at the outer edges and in the innercore inside the MB calyces, respectively, and class II KCs, whose somata are localized at the outer surface of the MB calyces [4–8]. Genetic studies in *Drosophila melanogaster* revealed that the MBs are involved in learning and memory [11–13]. In the honeybee, MBs function not only in learning and memory but also multimodal sensory integration [14–16].

Some preceding studies showed that the MB composition changes during the transition of workers from nurse bees to foragers as well as related to the foraging experience, implying that the MB function relates to the foraging behavior [17, 18]. In addition, Farris and Schulmeister demonstrated that during Hymenopteran evolution from a solitary lifestyle through a parasitic to a eusocial lifestyle MB elaboration is associated with the emergence of parasitism rather than sociality [19]. The authors proposed that the complex MB structure has been acquired associated with the foraging behaviors of parasitic wasps [19]. These studies suggest that the MB functions are related to foraging behaviors in the honeybees.

To identify the molecular and neural bases underlying advanced honeybee brain functions, we and other groups have searched for genes expressed in a brain area-preferential manner. So far, each KC subtype has been found to have a distinct gene expression profile in the honeybee brain, suggesting their distinct cell characteristics (e.g., [20–22], for review, see [23, 24]). The role of each KC subtype in honeybee social behaviors, however, is not well understood. We recently identified a novel KC subtype that we termed ‘middle-type’ KCs (mKCs), which are characterized by the preferential expression of a gene termed *mKast* (*m**iddle-type* *K**C preferential* *a**rre**st**in-related protein*) [25]. Although *mKast* was originally identified during the screening of genes whose expressions are more enriched in the OLs than in the other regions in the worker brain, detailed expression analysis revealed that *mKast* is also expressed in the MBs with a very unique expression pattern: it is preferentially expressed at the interface of the lKCs and sKCs in the worker MBs. Neural activity mapping using an immediate early gene, termed *kakusei* [26], revealed that both the sKCs and a part of mKCs are mainly active in the forager brains, suggesting the role of both KC subtypes in information processing during the foraging flight [25].

Our previous phylogenic tree analysis revealed that, although mKast contains both arrestin-like_N and arrestin-like_C domains, it is structurally not closely related to arrestins and rather has low (less than 30%) but significant sequence identities with mammalian arrestin-domain containing proteins (ARRDCs) [25]. Recent studies revealed that β-arrestin2 (or arrestin-3) and ARRDCs function sequentially to traffic the agonist-stimulated β_2_ adrenergic receptors (β2AR) to sorting endosomes [27–30]. There are two types of arrestins in mammals: visual and non-visual arrestins (e.g., β-arrestin2), which are selectively expressed in photoreceptor cells and mainly expressed in neurons, respectively [31, 32]. Although *mKast* encodes an arrestin-related protein, as the name indicates [25], its role in the honeybee brain remains totally unknown.

In the present study, as the first step to elucidate the functions of mKast in the honeybee brain and its possible relationship with honeybee social behaviors, we performed expression analysis of *mKast* to analyze its body part- and developmental stage-specific expressions. In addition, we performed immunohistochemical analysis using anti-mKast antibodies to examine localization of the mKast protein in the brains of worker honeybees.

## Methods

### Animals

European honeybee (*Apis mellifera* L.) colonies maintained at The University of Tokyo (Hongo campus) were used throughout the experiments. Some colonies were also purchased from a local dealer (Kumagaya Honeybee Farm, Saitama, Japan). Nurse bees and foragers were collected according to their behaviors and the degree of the hypopharyngeal gland development, as described previously [33]. Briefly, in-hive bees that inserted their heads into larval cells to feed the brood and had well-developed hypopharyngeal glands (exocrine glands that synthesize and secrete major royal jelly proteins) were collected as nurse bees. Workers that returned to their hives with pollen loads on their hind legs and had shrunk hypopharyngeal glands were collected as foragers.

### Preparation of recombinant mKast and anti-mKast antibody

An mKast cDNA fragment (+480 to +1757, NCBI RefSeq:XM_006564060.1), which contains the entire coding region of *mKast* [25], was amplified by polymerase chain reaction (PCR), using KOD-Plus DNA polymerase (Code No. KOD-201, TOYOBO, Japan) and a forward gene-specific primer containing a *Bam*H1 site and a reverse gene-specific primer containing an *Eco*R1 site for the *mKast* coding region. The primer sequences used were: 5’-TACGGATCCATGGAGGACG-3’ for the forward primer and 5’-CATGAATTCTTAAAGAGCCTTTTTC-3’ for the reverse primer (restriction enzyme sites are underlined). The PCR conditions used were: one cycle for 2 min at 94°C plus 30 cycles of 15 s at 94°C, 30 s at 58°C, and 2 min at 68°C. After double digestion with restriction enzymes, the cDNA fragment was subcloned into pET-22b(+) vector (Cat. No. 69744–3, Novagen, CA) with a pGEM-T Easy kit (Cat. A-1360, Promega, WI), and used to transform *Escherichia coli* BL21 (DE3). The cDNA for mKast inserted into the pET-22b(+) vector was sequenced and confirmed. After expression of the recombinant mKast protein with a 6 x His tag at the N-terminus (hereafter, recombinant mKast) was induced by incubating transformed *Escherichia coli* BL21 (DE3) in a medium containing 0.1M isopropyl β-D-1-thiogalactopyranoside (IPTG; Takara, Japan) at 37°C for 3 h, the cells were collected by centrifugation, and lysed with Lysis buffer (10mM Tris-HCl, pH 8.0, containing 8M urea, 100mM NaH_2_PO_4_ and 10mM imidazole and EDTA-free Protease Inhibitor Cocktail (cOmplete^™^; Roche, NJ)). The cells were lysed under denaturing conditions to increase the recovery of recombinant mKast. In addition, the lysis buffer contained Protease Inhibitor Cocktail to avoid possible degradation of the recombinant mKast. The recombinant mKast was isolated from the cell lysate by Ni-NTA affinity chromatography (Ni-NTA agarose; Cat. No. 30210, Qiagen, Japan), according to the manufacturer’s protocol.

The isolated recombinant mKast (total approximately 2.4mg) was subjected to SDS-polyacrylamide gel electrophoresis (PAGE), and the gel portion corresponding to the band for recombinant mKast was excised, homogenized and used to immunize guinea pigs. Anti-mKast antisera were obtained after two times of booster injection (these processes were performed by Evebio-science Co.Ltd., Wakayama, Japan). Next, anti-mKast antibodies were affinity-purified. First, after the purified recombinant mKast (approximately 190μg) was subjected to SDS-PAGE and blotted to polyvinylidene difluoride membrane (BIO-RAD, Japan), the membrane portion corresponding to recombinant mKast was excised and incubated with anti-mKast antisera in TBS-T (20mM Tris-HCl, pH 7.4, containing 150mM NaCl, 2mM EDTA-3Na and 0.1% Tween 20) at 4°C for overnight with shaking. After washing the membrane portion with TBS-T, bound antibodies were eluted with 0.2M glycine-HCl, pH 2.0, and the solution was adjusted to pH7.5 by adding 1.0M Trizma base (Sigma, MO). The antibody concentration was estimated based on the absorbance at 280nm.

### Western blotting and immunohistochemistry

After ten workers (a mixture of nurse bees and foragers) were anesthetized on ice, their brains were dissected from the heads under binocular microscopy and homogenized in 250μl lysis buffer (58mM Tris-HCl, pH 6.8, containing 70mM SDS and 50% (v/v) glycerol). Protein concentration was determined using a BCA Protein Assay Reagent Kit (PIERCE, IL). Western blotting was performed essentially as described previously [33], using 1μg/ml affinity-purified anti-mKast antibodies or the same concentration of normal guinea pig IgG (MBL) as the first antibody, and 1/2000-fold diluted horseradish peroxidase (HRP)-conjugated anti-guinea pig IgG antibody (Sigma, MO) as the second antibody. After the membrane was treated with a luminol chemiluminescent substrate (LumiGLo; Cell Signaling, Japan), the antigen was detected by chemiluminescence followed by autoradiography.

For immunohistochemistry, brains were dissected from the heads of workers and fixed in phosphate buffered saline (PBS) containing 4% paraformaldehyde at 4°C for 2h, followed by replacement with PBS containing 20% sucrose at 4°C for 20h. Then the brains were embedded in an O.C.T. compound (SAKURA Tissue-Tek, Japan) and rapidly frozen on dry ice, and 10 μm serial brain sections were prepared using a cryostat. After the brain sections were washed with PBS and PBS-Tx (PBS containing 0.1% Triton X-100) and blocked in 2% NGS/PBS-Tx (PBS-Tx containing 2% normal goat serum (NGS)) at room temperature for 1h, they were incubated with 2% NGS/PBS-Tx containing 20μg/ml affinity purified anti-mKast antibodies or normal guinea pig IgG, as a negative control, at room temperature for 1h. Then the brain sections were washed with PBS for three times, and incubated with 1/1000-fold diluted Alexa Fluor 546 anti-guinea pig IgG antibody (Thermo Fisher Scientific, MA) for 1h. After the brain sections were stained with 4',6-diamidino-2-phenylindole (DAPI, Sigma, MO), followed by washing with PBS for three times, they were observed using fluorescent microscopy (ZEISS Axio Imager Z1, ZEISS, Japan). Serial 10μm brain sections were alternately used for immunohistochemical staining with affinity purified anti-mKast antibodies or normal guinea pig IgG as a negative control, respectively, to precisely detect antibody-specific signals.

### Quantitative reverse transcription polymerase chain reaction (qRT-PCR)

**Fig 1:**
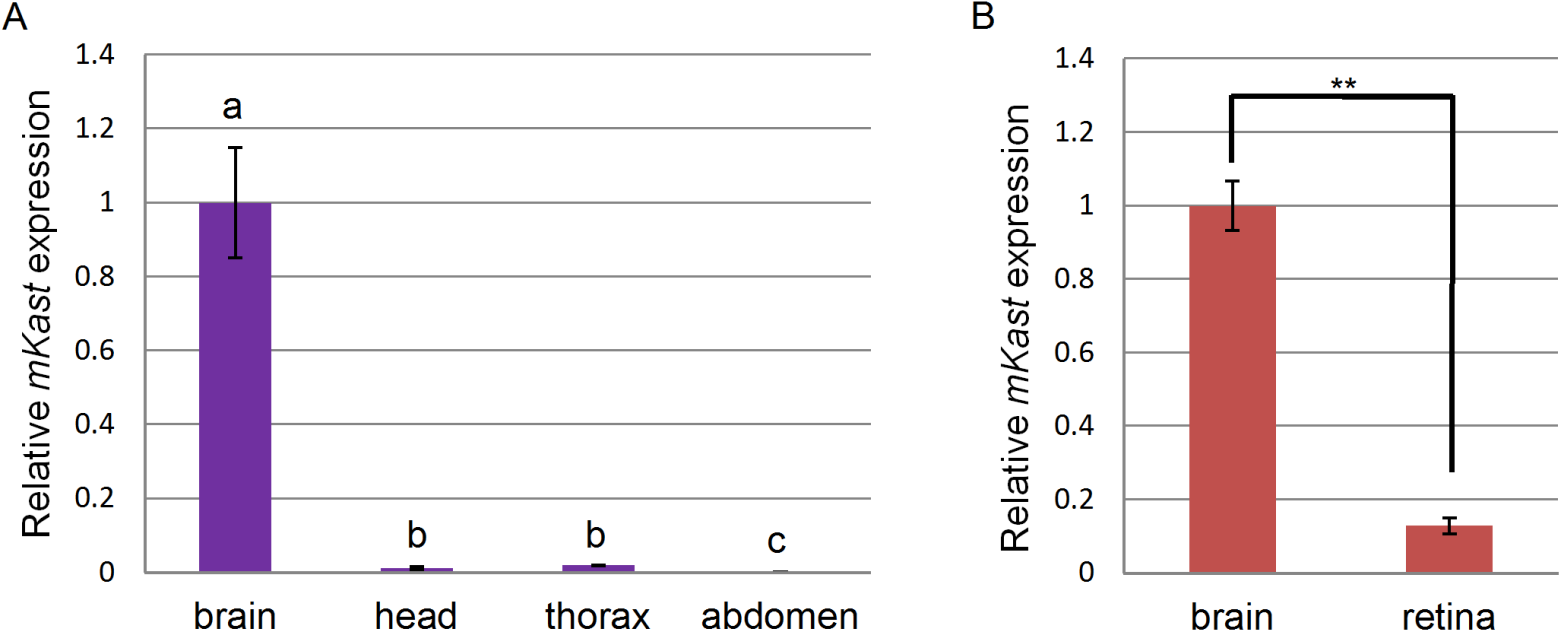
qRT-PCR analysis of *mKast* expression in various worker body parts. (A) The amounts of *mKast* transcript in the worker brains, heads without brains, thoraxes and abdomens were determined by qRT-PCR and normalized with that of *Rpl32* transcript. The Steel-Dwass test was used for statistical analysis. Different letters (a, b and c) indicate statistical differences (p < 0.01). Data are shown as mean ± SEM (n = 7). (B) The amounts of *mKast* transcript in the worker brains and retinas were determined by qRT-PCR and normalized with that of *Rpl32* transcript. Welch’s t-test was used for statistical analysis. Data with a significant difference are indicated with ** (p < 0.01). Data are shown as mean ± SEM (n = 5).

**Fig 2:**
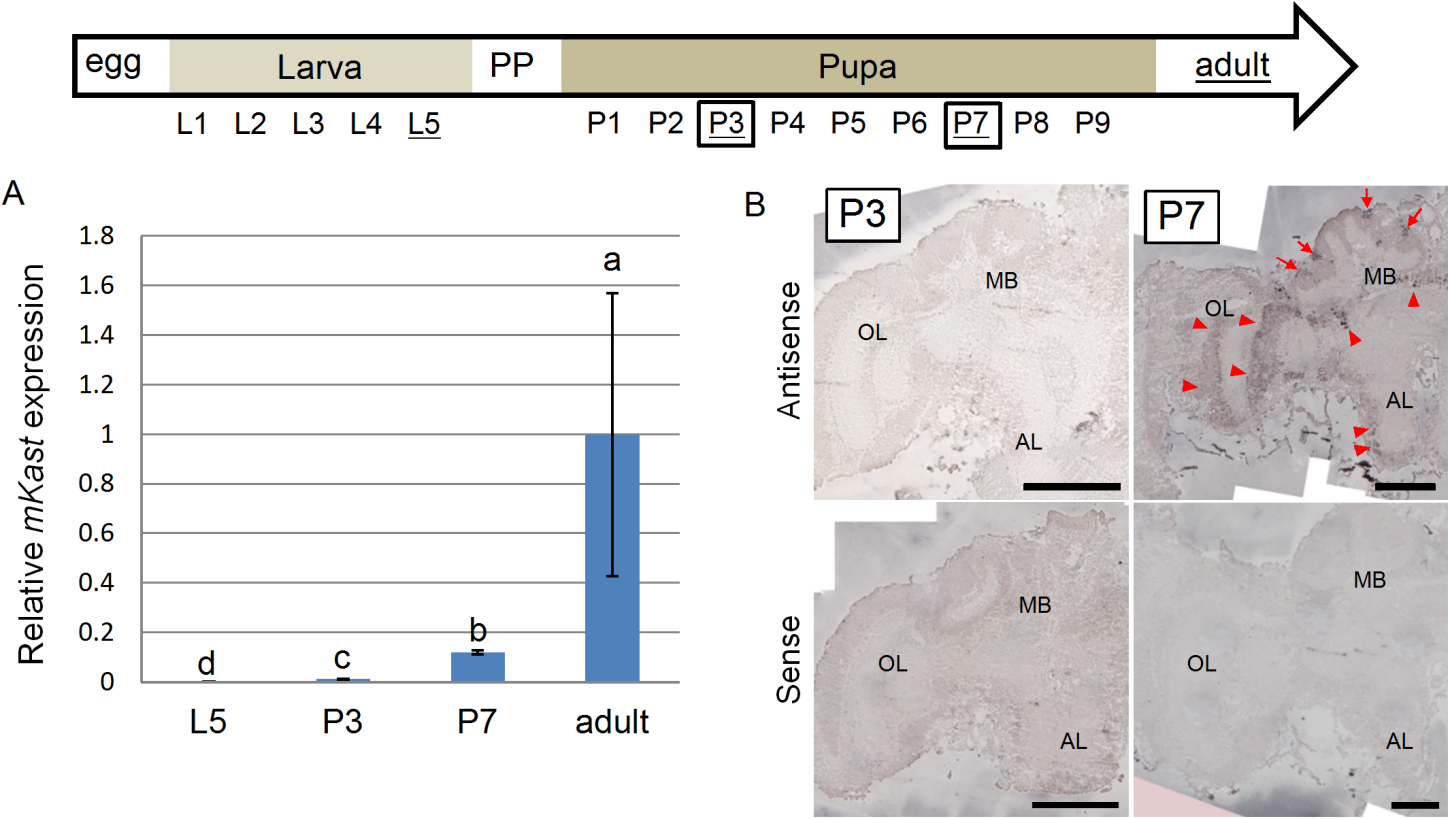
qRT-PCR and *in situ* hybridization analyses of *mKast* expression in pupal brains during metamorphosis. Upper arrow indicates progression of the developmental stages of workers from egg (left) through larva, prepupa (PP), pupa to adult (right). L1 to L5 indicate first to fifth larval instar, respectively. P1 to P9 indicate pupal stages: 1 to 9 days after puparium formation, respectively. Developmental stages analyzed by *in situ* hybridization (P3 and P7) are boxed. (A) The amounts of *mKast* transcript in the brains of workers at different developmental stages (L5, P3, P7 and adult) were determined by qRT-PCR and normalized to *gapdh* transcript amounts. The Steel-Dwass test was used for statistical analysis. Different letters (a, b and c) indicate statistically significant differences (p < 0.05). Data are shown as mean ± SEM (for L5, P3, P5 and adult samples, n = 6, 5, 5 and 7, respectively). (B) *In situ* hybridization analysis of *mKast* in the brains of pupae at the P3 and P7 stages. Upper and lower panels indicate results with antisense and sense probes, respectively. Left and right panels indicate brains of pupae at the P3 and P7 stages, respectively. *mKast* signals are detectable in several brain regions of pupa at the P7 stage with antisense probe. Red arrows indicate signals at the MB mKCs and red arrowheads indicate signals in the other brain regions. MB, mushroom body; OL, optic lobe; AL, antennal lobe. Scale bars indicate 200 μm. Note that retina is removed from our specimen. There is a huge variance in adult brain mKast levels, because *gapdh* expression, which was used to normalize *mKast* expression, for one of the seven adult brain samples was very small. The raw data for quantification of *mKast* and *gapdh* transcripts of the seven samples are provided in S2 Table.

Larvae, pupae and workers were collected from the same colony and the developmental stages of larvae and pupae were evaluated as described previously [25]. After the bees were anesthetized on ice, brains and the other body parts (heads without brains, thoraxes without legs and wings, abdomens and retinas) were dissected with fine scissors in insect saline (10mM Tris-HCl, pH 7.4, containing 150mM NaCL, 5mM KCl and 1mM CaCl_2_) under a binocular microscope, and homogenized in TRIzol reagent (Thermo Fisher Scientific, MA) with a bead cell crasher (MS-100; Tomy, Japan) or micropestles. One sample (brain, head, thorax or abdomen) was collected for each lot and seven lots were used for comparison between adult body parts (Fig 1A); three samples (brain or retina) were collected for each lot, because the volumes of the brains and retinas are small, and five lots were used for comparison between brains and retinas (Fig 1B); one sample (L5, P3, P5 or adult brain) was collected and six (L5), five (P3), five (P5), and seven lots (adult brain) were used for the comparison between the metamorphosis stages (Fig 2). The same samples were used as ‘brain’ for the comparison with adult body parts and as ‘adult’ for the comparison with metamorphosis stages (S1 Table).

qRT-PCR was performed essentially as described previously [25], using a mixture of oligo dT primers and random hexamers. The amount of total RNA of each lot used for the reverse transcription was usually 500 ng or 1000 ng, except in some cases, in which 413, 431, and 484 ng of total RNA were used (S1 Table). In all qRT-PCR experiments, only one set of samples was analyzed. Total RNA extracted using TRIzol reagent was reverse transcribed with PrimeScript RT reagent kit with gDNA eraser (Takara, Japan). qRT-PCR was performed with LightCycler (Roche, NJ) using SYBR Premix Ex Taq II (Takara, Japan) and forward and reverse gene specific primers for *mKast*; 5’-TCCAGCAGTACCGTTGTACG-3’ and 5’-CGAGTACGGCTTGACCTCTC-3’, for *Ribosomal protein L32* (*RpL32*); 5’-AAAGAGAAACTGGCGTAAACC-3’ and 5’-CAGTTGGCAACATATGACGAG-3’, for *gapdh*; 5’-GATGCACCCATGTTTGTTTG-3’ and 5’-TTTGCAGAAGGTGCATCAAC-3’, respectively. The amount of *mKast* transcript was normalized with that of *RpL32* for the analysis of organ-specific *mKast* expression, and with that of *gapdh* for the analysis of developmental stage-specific *mKast* expression, because the amount of *gapdh* transcript less varied compared to that of *RpL32* during pupal stages, while that of *RpL32* considerably varied (data not shown). Welch’s t test was used for the expression analysis of *mKast* among worker brains and retinas, and Steel-Dwass test was used for organ-specific and developmental stage-specific expression analyses.

### *In situ* hybridization

*In situ* hybridization was performed as described previously [25]. Briefly, frozen vertical pupal brain sections (10 μm thick) were fixed in 4% paraformaldehyde in 100 mM sodium phosphate buffer, pretreated, and hybridized with digoxigenin (DIG)-labeled riboprobes. The template plasmid (GenBank accession no. BP874957) contains 754-bp nucleotide sequences that correspond to an exon and 3’-UTR of mKast cDNA [25]. The DIG-labeled sense and antisense RNA probes were prepared by *in vitro* transcription of the above template plasmid using a DIG RNA labeling kit (Roche, NJ). After stringent washes, DIG-labeled riboprobes were detected immunocytochemically with alkaline phosphatase-conjugated anti-DIG antibody (1:500; Roche, NJ). Sense probes were used as negative controls and the signals were confirmed to be antisense probe-specific in every experiment. Nuclei were stained with DAPI.

## Results

### Analysis of body part- and developmental stage-specific *mKast* expression

To elucidate the possible functions of mKast in the honeybee, it is important to understand its spatial (organ-specific) and temporal (developmental stage-specific) expression profiles. Although we previously used *in situ* hybridization analysis to analyze brain region-preferential *mKast* expression in the worker brains, we have not analyzed its expression in the other body parts. Therefore, we first performed qRT-PCR using total RNAs extracted from the brains, heads without brains, thoraxes without legs and wings, and abdomens. *mKast* expression was most prominent in the brains, whereas it was scarcely detected in the other body parts (Fig 1A, p<0.01), suggesting that mKast mainly functions in the brain.

We next compared *mKast* expression levels in the brains and retinas of workers, because there are two types of arrestins in mammals: visual and non-visual arrestins (e.g., β-arrestin2) [31, 32]. *mKast* expression was approximately 10-fold higher in the brains than in the retinas (Fig 1B, **: p<0.01), suggesting that mKast mainly functions in brain neurons rather than in retinas. There seemed to be no significant retinal contamination in the brain samples, because the retina is dark red in color and can easily be discriminated from brain tissue. On the other hand, there are no appropriate genes expressed preferentially in the brain, but not in the retina. Nonetheless, we think we can say that the expression level of mKast is higher in the brain than in the retina.

Our previous *in situ* hybridization analysis revealed that *mKast* expression in the MBs starts at the late (P7) pupal stage [25]. We have not, however, quantitatively investigated *mKast* expression in the pupal brains during metamorphosis. Therefore, we performed qRT-PCR using total RNAs extracted from the whole brains of larvae (L5 stage), pupae (P3 and P7 stages) and adults, respectively. Although *mKast* expression was scarcely detected in the larval (L5) brains, it clearly increased at the late pupal (P7) stage (Fig 2A), coinciding with our previous report [25]. Here, we reexamined our previous *in situ* hybridization results, because we previously reported *mKast* expression only in the pupal MBs but not in the other brain regions [25]. *mKast* expression was detectable in some brain regions, including the OLs and ALs, in the brains of pupa at the P7 stage, while almost no significant expression was detected in the brains of pupa at the P3 stage (Fig 2B), which coincides with the present qRT-PCR result (Fig 2A), strongly suggesting that mKast functions in the adult brain but not in the larval brain.

### Immunoblotting analysis of the worker brain homogenate with anti-mKast antibodies

**Fig 3:**
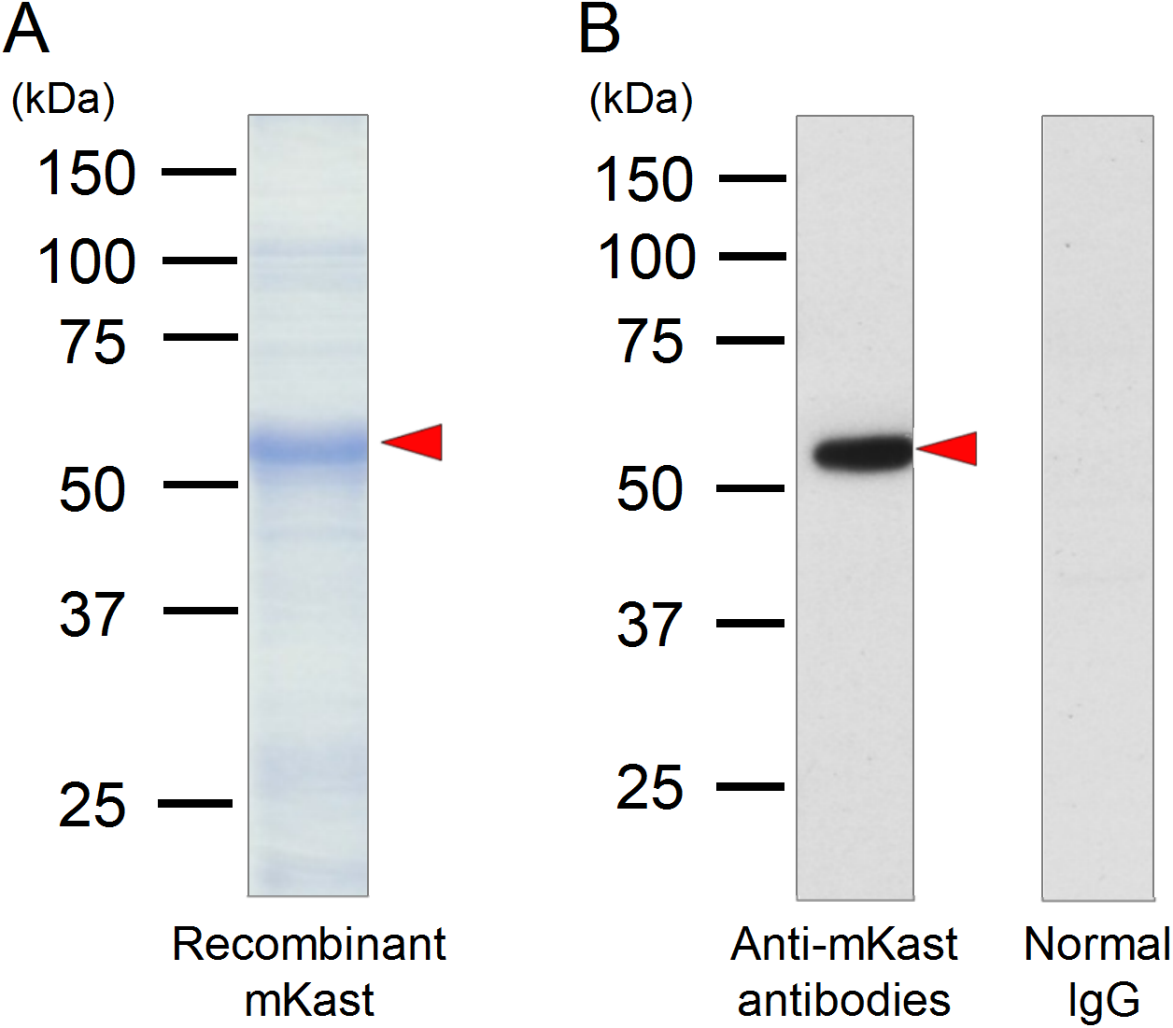
Immunoblotting analysis with affinity purified anti-mKast antibodies. (A) SDS-polyacrylamide gel electrophoresis of the purified recombinant mKast protein. The band corresponding to recombinant mKast is indicated by a red arrowhead. The positions of molecular mass markers are indicated at the left of the lane with kDa. (B) Immunoblotting analysis of the worker brain homogenates using affinity purified anti-mKast antibodies (left) or normal IgG (right). The band corresponding to mKast is indicated by a red arrowhead.

Since *mKast* was prominently and specifically expressed in the adult worker brain, we next intended to analyze localization of the mKast protein in the worker brains by immunohistochemistry using specific anti-mKast antibodies. When the isolated recombinant mKast was subjected to SDS-PAGE, a band of approximately 55kDa was detected as a major band (Fig 3A). As there were some other bands besides the band of 55kDa, we excised the gel portion corresponding to the recombinant protein of 55kDa and used it to prepare antisera in guinea pigs. These antibodies were affinity-purified from the antisera. When the homogenate of worker brains was subjected to immunoblotting analysis using the affinity-purified antibodies, a single band of 55kDa, which coincides well with the molecular weight of the recombinant protein of 55kDa, was detected, while no signal was detected with normal guinea pig IgG (Fig 3B).

Although there was a discrepancy in molecular weights between the predicted value for mKast (47.4kDa) and that observed in SDS-PAGE/immunoblotting (55kDa), we concluded that the 55kDa band corresponds to mKast based on the following evidence: 1) The mKast cDNA inserted into the expression vector was sequenced and confirmed, 2) the intensity of the 55kDa band drastically increased in the cytosol fraction of transformed *E*. *coli* after induction by 0.1M IPTG (data not shown) and 3) bands with the same molecular weight of 55kDa were detected in both SDS-PAGE and immunoblotting. These results indicated that the expressed protein of 55kDa actually exists in the worker brain homogenate and the anti-mKast antibodies specifically detected mKast-like-immunoreactivity.

### Immunohistochemical analysis of mKast in the worker brains

**Fig 4:**
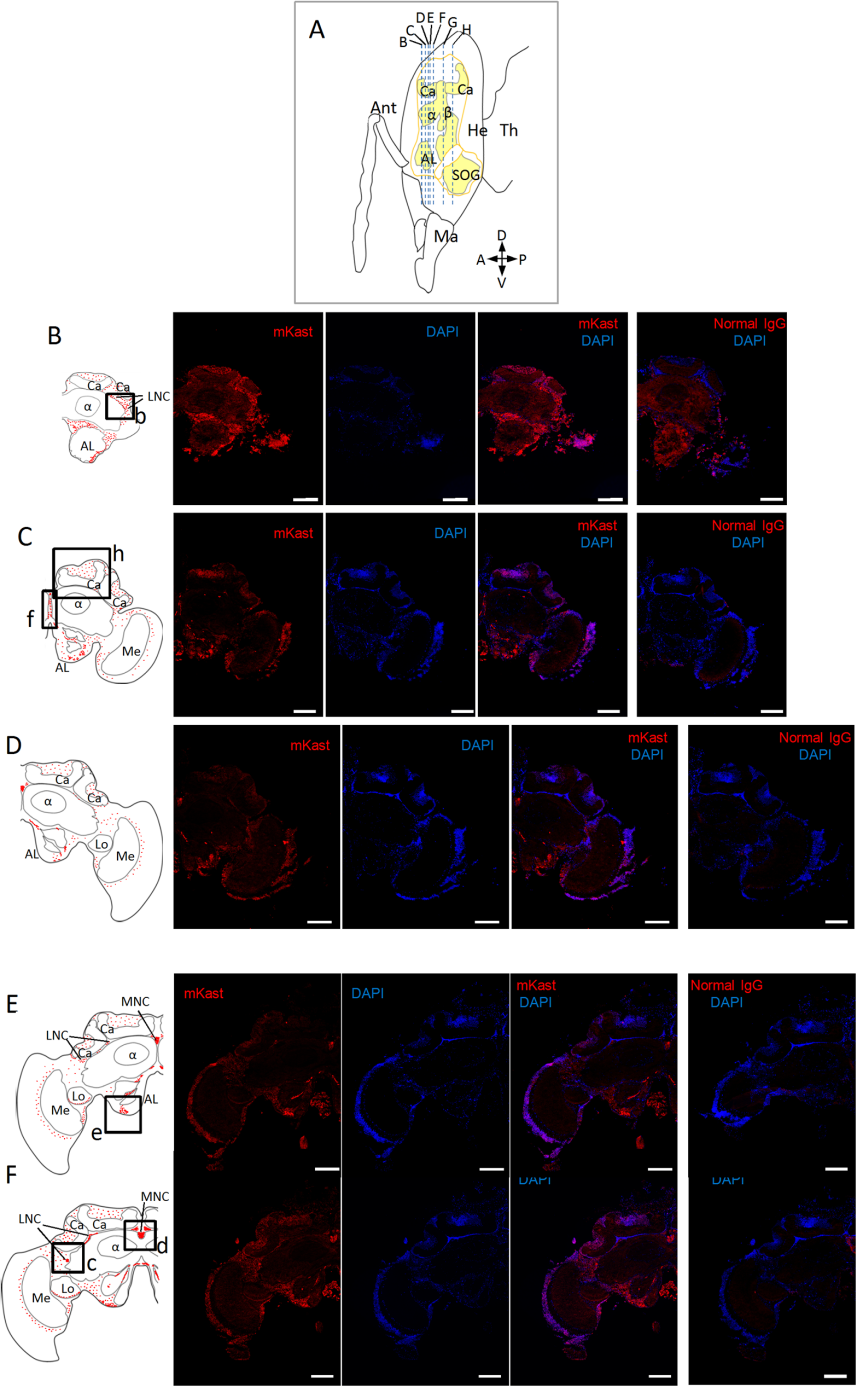
Fluorescent immunohistochemical analysis of worker brain sections using anti-mKast antibodies. (A) Schematic drawing of a worker head (sagittal view). Outline of the brain is indicated by orange lines. Areas that comprise neuropiles are shown in yellow. Blue broken vertical lines indicate location of coronal brain sections B-H. Ant, antenna: Ma, mandible; He, head; Th, thorax. A, P, D and V indicate anterior, posterior, dorsal and ventral, respectively. (B-H) Photomicrographs of immunohistochemical sections at different depth B-H. Left panels; schematic drawings of the distribution of mKast-like immunoreactivity (red dots) in the coronal brain sections B-H indicated in (A). Boxes a-i, see below. Ca, MB calyx; α, MB vertical lobe; β, MB medial lobe; La, lamina; Me, medulla; Lo, lobula; CB, central body; AL, antennal lobe; SOG, subesophageal ganglion; MNC, medial neurosecretory cell; LNC, lateral neurosecretory cell. Photomicrographs from left to right; serial 10μm coronal brain sections stained using affinity purified anti-mKast antibodies (leftmost photos), the same section stained with DAPI (2nd photos), merged images of antibody-staining and DAPI-staining (3rd photos), and the serial sections stained with normal guinea pig IgG and DAPI as a negative control (right panels), respectively. Red and blue signals indicate mKast-like immunoreactivity analyzed with anti-mKast antibodies and nuclear staining with DAPI, respectively. (a-i) Magnified views of merged images of antibody-staining and DAPI-staining (3rd photos of the above photomicrographs) of brain regions corresponding to boxes a-i shown in the left schematic drawings. mKast-like immunoreactivities detected at the outer surface of the OL lobula (a), LNC somata localized beneath the lateral MB calyx (b and c), MNC somata localized between the medial MB calyces (d), somata of neurons located at the outer surface of the ALs (e), putative projections to ALs (f), somata of neurons localized at the outer surface of SOG (g), inside of the MB calyces (h and i), are indicated by white arrowheads. Red and blue signals indicate mKast-like immunoreactivities and nuclear staining with DAPI, respectively. mKast-like immunoreactivities detected at the outer surface of the OL lobula are surrounded by a white broken line (a). Scale bars indicate 200 μm in panels (B-H) and 100 μm in panels (a-i), respectively. Note that the DAPI signals are weak in Fig 4B, probably because DAPI staining was not enough in these panels. Also note that only representative positive signals are indicated by white arrowheads in panels (a-i), because many cells with positive signals are clustered in these panels.

Then we performed immunohistochemical analysis using the affinity purified anti-mKast antibodies, or normal guinea pig IgG as a control, and 10μm frozen sections of the worker brains. When coronal sections that correspond to the anterior to posterior regions of the brains were used, mKast-like immunoreactivities were detected in some regions of the honeybee brain (Fig 4).

In coronal sections corresponding to the anterior brain regions, which contained the ALs but not SOG, mKast-like immunoreactivities were detected in some brain regions: while moderate mKast-like immunoreactivities were detected inside the MB calyces (Fig 4B–4F and 4H), strong mKast-like immunoreactivities were detected beneath the lateral MB calyces where the somata of lateral neurosecretory cells (LNCs) reside (Fig 4B, 4E, 4F, 4B and 4C), between the medial MB calyces where the somata of medial neurosecretory cells (MNCs) are located (Fig 4D–4F and 4D) [34], as well as at the outer surface and posterior of the ALs (Fig 4B–4F and 4E). In some sections, mKast-like immunoreactivities were also detected on the midline between the MB α lobes (Fig 4C and 4F), which might correspond to some projections to ALs [35].

In coronal sections corresponding to the posterior brain regions, which contained major parts of the OLs and SOG but not ALs, moderate mKast-like immunoreactivities were again detected inside the MB calyces (Fig 4G and 4H and 4I) and strong mKast-like immunoreactivities were detected in the MNCs (Fig 4G and 4H). In addition, clear mKast-like immunoreactivities were detected at the OL medulla neurons (Fig 4C–4G), outer edges of OL lobula (Fig 4E–4H and 4A) and at the outer surface of SOG (Fig 4G, 4H and 4G). No significant staining were detected in any brain areas with normal guinea pig IgG, indicating that all the above staining represented mKast-like immunoreactivity. In summary, mKast-like immunoreactivity was detected in several brain regions, including the MBs, OLs, ALs and SOG.

### Reexamination of *mKast* expression by *in situ* hybridization

**Fig 5:**
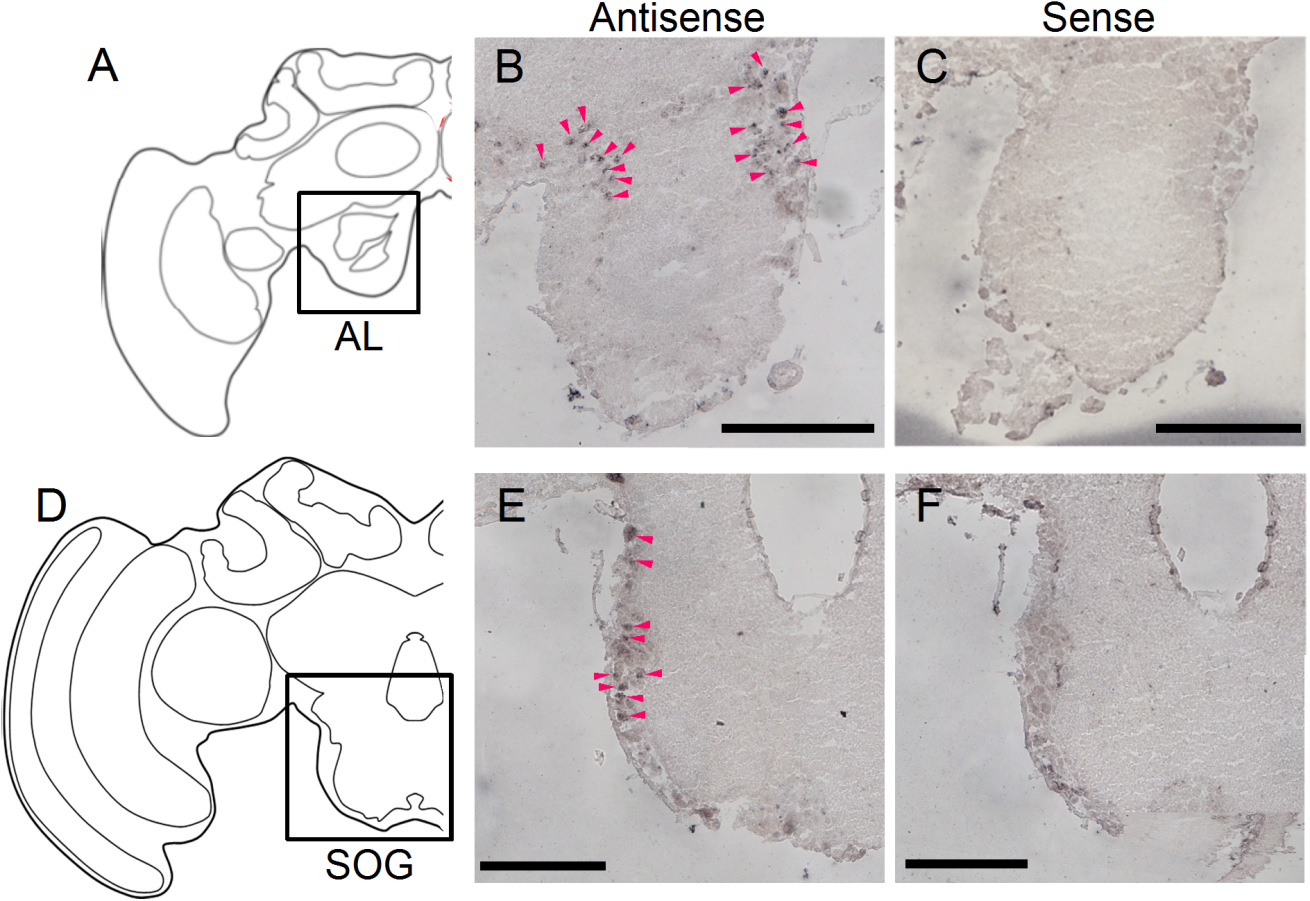
*In situ* hybridization analysis of *mKast* in adult worker brain. (A and D) Schematic drawings of the anterior and posterior coronal brain sections used for *in situ* hybridization analysis. (B, C) and (E, F) Magnified images of the results of *in situ* hybridization analysis of *mKast* that correspond to the boxed areas in (A) and (D) with antisense (B and E) or sense probes (C and F). Note that the sections in (A-C) contain regions corresponding to the posterior ALs and anterior SOGs, whereas the sections in (D-F) contain regions corresponding to SOGs. Red arrowheads in panels (B) and (E) indicate *mKast* expression. AL, antennal lobe; SOG, subesophageal ganglion. Scale bars indicate 200 μm.

Although we previously reported that *mKast* expression is enriched in the OLs as well as in the MB mKC [25], we did not focus on expressions around the ALs and SOG. However, since strong mKast-like immunoreactivities were detected at the outer surface and posterior of the ALs (Fig 4B–4F and 4E) as well as at the outer surface of the SOG (Fig 4G, 4H and 4G) in the present study, which suggests that mKast is localized in both the ALs and SOG, we reexamined by *in situ* hybridization whether *mKast* is expressed in the ALs and SOG in the worker brain. Significant signals were also detected in the cell body layer of the dorsal lobe / tritocerebrum posterior to the ALs (Fig 5A–5C) as well as the outer surface of the SOG (Fig 5D–5F), which is also consistent with our present results of the mKast immunohistochemistry.

## Discussion

In mammals, arrestins and ARRDCs function in a hierarchical manner to traffic agonist-stimulated G protein-coupled receptors (GPCRs) to sorting endosomes [27–30]. For example, while **β**-arrestin2 (arrestin-3) is the primary adaptor that binds agonist-stimulated β2AR to promote clathrin-dependent internalization, ARRDCs are secondary adaptors that bind internalized β2AR complexes on endosomes to traffic them to a subpopulation of early endosomes [29, 30]. While visual arrestins regulate rhodopsin functions in photoreceptor cells, non-visual arrestins and ARRDCs regulate functions of various GPCRs mainly in neural tissues [31, 32]. In the present study, we demonstrated that *mKast* expression was almost entirely restricted to the brain among the various worker body parts (Fig 1A), and more enriched in the brain than in the retina (Fig 1B). Therefore, it is plausible that mKast regulates functions of some GPCRs in the worker brain, like mammalian ARRDCs.

*mKast* expression started at the late (P7) pupal stage in the whole brain, whereas it is scarcely expressed at the early pupal (P3) stage (Fig 2A), which coincides well with our previous results that *mKast* expression becomes detectable in the mKCs at the P7 stage by *in situ* hybridization [25]. As proliferation of lKCs and sKCs cease at the P3 and P5 stages, respectively [36], we previously discussed that the mKC lineage may newly arise from the lKC or sKC lineage or both of them by acquisition of distinct cell characteristics [25]. Interestingly, *mKast* expression started not only in the MB mKCs but also in the other brain regions, including the OLs and ALs, at the P7 stage (Fig 2B), which is late compared to three other genes that are expressed preferentially in the lKCs in worker brains and begin to be expressed at the P2 stage; i.e., *Mblk-1*, *Syt14*, and *dlg5* [37], or two other genes that are strongly expressed in the OLs in worker brains and begin to be expressed at the P2 stage; i.e., *futsch* and *Tau* [38]. These results suggest that *mKast*-expression represents the molecular characteristics of some MB, AL, OL and SOG neurons at their late differentiation stages.

Immunohistochemical analyses revealed that mKast-like immunoreactivity is distributed in some restricted brain regions: while moderate mKast-like immunoreactivities were detected inside the MB calyces (Fig 4B–4H, 4H and 4I), strong mKast-like immunoreactivities were detected at the outer margins of the OL lobula and around the medulla (Fig 4E–4H and 4A), inner and outer surfaces of the ALs (Fig 4B–4F and 4E), and outer surface of the SOG (Fig 4G, 4H and 4G). Although *mKast* is preferentially expressed in the MB mKCs [25], mKast-like immunoreactivity was localized uniformly inside the MB calyces. There are some possible explanations for the differences in localization of the *mKast* transcript and mKast protein: it is possible that, while mKast is also expressed in the lKCs and sKCs at the basal level, mKast prominently expressed in the mKCs might be rapidly degraded in the mKC somata, and thus the preferential localization of mKast-like immunoreactivity in the mKCs was not observed. Alternatively, it is also possible that mKast prominently expressed in the mKCs is rapidly transported to the other brain regions through the MB dendrites or axons.

The honeybee OLs comprise three neuropils, termed lamina, medulla and lobula, from distal to proximal [10, 39–41]. Contrast of light and darkness, and local motion are mainly processed in lamina and medulla, respectively, whereas wide-field motion is processed in the distal lobula [42, 43]. Considering that most of the neurons located at the medulla-lobula layer express *mKast* [25] and that mKast-like immunoreactivity was mainly localized at the outer edges of OL lobula (Fig 4E–4H and 4A), it is plausible that mKast plays important roles in these reurons. mKast-like immunoreactivity was also detected in neurons localized at the outer surfaces and behind the ALs (Fig 4B–4F and 4E) as well as at the outer surface of SOG (Fig 4G, 4H and 4G), suggesting their functions in the ALs and SOG. The signals detected at the surface of SOGs may contain octopamine-positive neurons [35]. Although no prominent *mKast*-expression was detected in the ALs and SOGs in our previous study [25], we detected strong mKast-like immunoreactivity in both the ALs and SOGs, possibly because the translation efficiency of mKast protein is higher, or the degradation speed of mKast protein is smaller in the ALs and SOGs than in the MBs and OLs.

mKast-like immunoreactivity was also detected in the LNCs and MNCs that are located beneath and between the MB calyces (Fig 4B, 4D–4H and 4B–4D). In insects, NCs project to the corpus cardiacum and the corpus allatum to regulate secretion of some neurotransmitters and juvenile hormone [34, 35]. It might be that mKast also regulates functions of receptors that are expressed in the LNCs and MNCs. Strong mKast-like immunoreactivities detected in the ALs, SOG as well as in the MNCs and LNCs may partly reflect their larger cell bodies, or represent their higher translation efficiencies of mKast compared to those in the KCs or OL neurons. Finally, whether the cells with mKast-like immunoreactivity are neurons or glial cells requires further evaluation in future studies.

Recent studies classified the arrestin family into visual-arrestins, β-arrestins (e.g., β-arrestin2), and α-arrestins (ARRDCs in mammals) [44]. Although α-arrestins are mainly membrane-associated proteins, recent studies indicated that they change their subcellular localization in response to different stimuli [44]. For example, TXNIP, a member of mammalian α-arrestin, interacts with importin-α to shuttle into the nucleus [45], and a part of α-arrestin 1 (ARRDC1) is localized at the plasma membrane, whereas another part is co-localized with cytoplasmic vesicles [46]. ARRDCs are localized on the inner sides of plasma membrane, lysosomes and endosomes [46]. Interestingly, most of the mKast-like-immunoreactivity was detected in the cell bodies rather than in the neuropils in the MBs, OLs, ALs, SOGs, MNCs and LNCs (Fig 4). Supposing that mKast also functions as a member of α-arrestin, these results suggest that a large part of mKast protein is continuously detached from the plasma membrane of neurons and/or neurosecretory cells in these brain areas and localized with lysosomes and /or endosomes, or in the nuclei, in response to continuous pre- and/or post-synaptic stimuli to downregulate some receptor signaling. If this is the case, target receptors of mKast may have relatively short half-life on the plasma membrane.

The stimuli to which mKast might respond to retain its subcellular localization in the cell bodies remains unclear. Recent studies also demonstrated that α-arrestins act as scaffold proteins, not only in GPCRs but also in Notch signaling [44, 47], the latter of which plays a crucial role in fate decisions of a variety of cells, including neurons, in various animals [48, 49]. α-arrestin 1 and β-arrestins cooperate to promote non-activated Notch receptor degradation, which negatively regulates Notch signaling in mammals [47]. Supposing that mKast is involved in the regulation of Notch signaling even in honeybee brains, mKast, which begins to be expressed at the late pupal stage, might function to modify the fate of cells that express Notch. It may be interesting to investigate the possibility that modulation of Notch signaling by mKast triggers mKC differentiation at the P7 stage.

To test these possibilities, it might be important to identify target receptors of mKast in the honeybee brain. It will also be important to analyze mKast function using reverse genetic methods, such as CRISPR-Cas9 system [50], which has recently become available in the honeybee [51]. The extensive genome information available in the honeybee [52] will benefit the research strategies. Adult brain specific *mKast* expression may also benefit the functional analysis of *mKast* in the adult honeybees, because knocking out *mKast* might not cause embryonic lethality.

## Supporting information

S1 Table The amount of total RNA of each lot used for the reverse transcription. (XLSX)

S2 Table The quantified value of each samples for comparison of relative *mKast* expression between metamorphosis stages. (XLSX)
