# Correction: Gene expression and immunohistochemical analyses of mKast suggest its late pupal and adult-specific functions in the honeybee brain

**DOI:** 10.1371/journal.pone.0183522

**Published:** 2017-08-14

**Authors:** Atsuhiro Yamane, Hiroki Kohno, Tsubomi Ikeda, Kumi Kaneko, Atsushi Ugajin, Toshiyuki Fujita, Takekazu Kunieda, Takeo Kubo

Fig 4 is incorrect. The authors have provided a corrected version here.

**Fig 4:**
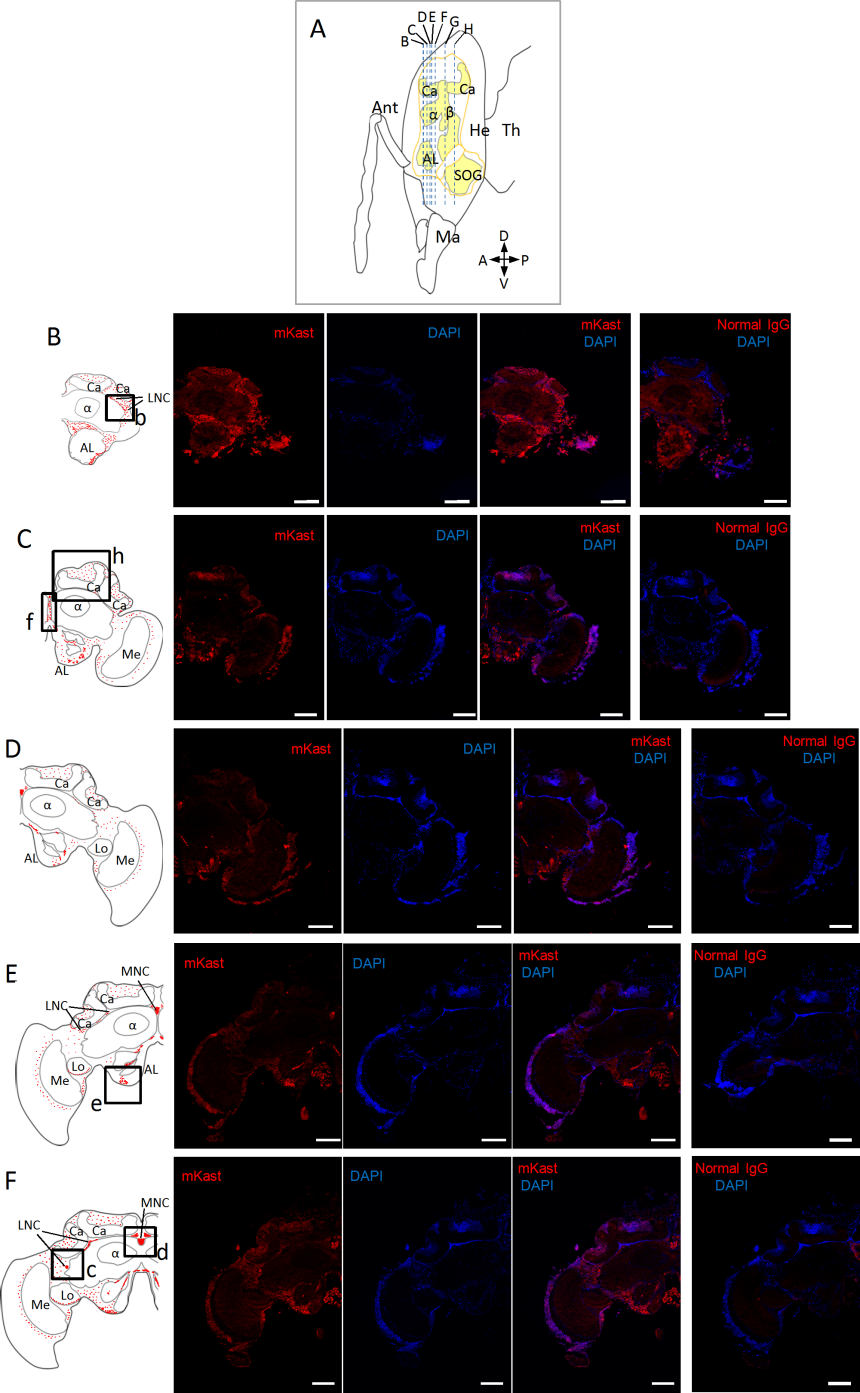
Fluorescent immunohistochemical analysis of worker brain sections using anti-mKast antibodies. (A) Schematic drawing of a worker head (sagittal view). Outline of the brain is indicated by orange lines. Areas that comprise neuropiles are shown in yellow. Blue broken vertical lines indicate location of coronal brain sections B-H. Ant, antenna: Ma, mandible; He, head; Th, thorax. A, P, D and V indicate anterior, posterior, dorsal and ventral, respectively. (B-H) Photomicrographs of immunohistochemical sections at different depth B-H. Left panels; schematic drawings of the distribution of mKast-like immunoreactivity (red dots) in the coronal brain sections B-H indicated in (A). Boxes a-i, see below. Ca, MB calyx; α, MB vertical lobe; β, MB medial lobe; La, lamina; Me, medulla; Lo, lobula; CB, central body; AL, antennal lobe; SOG, subesophageal ganglion; MNC, medial neurosecretory cell; LNC, lateral neurosecretory cell. Photomicrographs from left to right; serial 10μm coronal brain sections stained using affinity purified anti-mKast antibodies (leftmost photos), the same section stained with DAPI (2nd photos), merged images of antibody-staining and DAPI-staining (3rd photos), and the serial sections stained with normal guinea pig IgG and DAPI as a negative control (right panels), respectively. Red and blue signals indicate mKast-like immunoreactivity analyzed with anti-mKast antibodies and nuclear staining with DAPI, respectively. (a-i) Magnified views of merged images of antibody-staining and DAPI-staining (3rd photos of the above photomicrographs) of brain regions corresponding to boxes a-i shown in the left schematic drawings. mKast-like immunoreactivities detected at the outer surface of the OL lobula (a), LNC somata localized beneath the lateral MB calyx (b and c), MNC somata localized between the medial MB calyces (d), somata of neurons located at the outer surface of the ALs (e), putative projections to ALs (f), somata of neurons localized at the outer surface of SOG (g), inside of the MB calyces (h and i), are indicated by white arrowheads. Red and blue signals indicate mKast-like immunoreactivities and nuclear staining with DAPI, respectively. mKast-like immunoreactivities detected at the outer surface of the OL lobula are surrounded by a white broken line (a). Scale bars indicate 200 μm in panels (B-H) and 100 μm in panels (a-i), respectively. Note that the DAPI signals are weak in Fig 4B, probably because DAPI staining was not enough in these panels. Also note that only representative positive signals are indicated by white arrowheads in panels (a-i), because many cells with positive signals are clustered in these panels.
